# Synergistic Effects of Plasticizer Types on the Mechanical, Thermal, and Morphological Properties of PVC Compounds for Cable Application

**DOI:** 10.3390/polym18162015

**Published:** 2026-08-19

**Authors:** Furkan Kaya, Aysun Ekinci-Tekin, Mustafa Oksuz, Murat Ates

**Affiliations:** 1 Unal Cable, Velimese OSB, 247th Street, Number: 4/1, Ergene 59930, Turkey; furkankaya581@gmail.com; 2 Department of Chemistry, Faculty of Science, Nigde Omer Halisdemir University, Nigde 51100, Turkey; aysun.tekin@ohu.edu.tr; 3 Faculty of Engineering and Architecture, Recep Tayyip Erdogan University, Rize 53100, Turkey; 4 Department of Polymer Materials Engineering, Faculty of Engineering, Yalova University, Yalova 77200, Turkey; 5 Department of Chemistry, Faculty of Arts and Sciences, Tekirdag Namik Kemal University, Degirmenalti Campus, Tekirdag 59030, Turkey; mates@nku.edu.tr; 6 Nanochem Polymer Energy Company, Silahtaraga Mah., University 1st Street, Number: 13/1 Z242/1, Tekirdag 59860, Turkey

**Keywords:** plasticizer, PVC, cable, DOA, DOTP, TOTM

## Abstract

Poly (vinyl chloride) (PVC) is widely used in many products due to its increased flexibility and processability. It is preferred in many industrial applications, especially in the plasticized PVC cable industry due to its excellent insulation properties. PVC is quite hard and can be difficult to process. Therefore, it requires additives such as plasticizers. Plasticizers typically reduce the glass transition temperature (Tg) and provide flexibility by reducing the workable temperature level. In the PVC compound production industry, phthalate-based plasticizers are preferred due to their low cost. Commonly used plasticizers are adipates, azelates, trimethylates, phthalates, benzoates, and chlorinated paraffins. The aim of the study was to investigate the plasticizer changes in PVC compounds used in cable insulation applications by synergistic effects of adipate, trimellitate, and phthalate-based plasticizers such as dioctyl terephthalate (DOTP), 2-ethyl hexyl adipate (DOA), and tris(2-ethylhexyl) benzene-1,2,4-tricarboxylate (TOTM). In this study, the effects of plasticizer additives were investigated on the structural, morphological, thermal, and mechanical properties of PVC compounds in the cable industry. Fabricated test products were characterized using characterization methods such as Fourier transform infrared-attenuated total reflectance (FTIR-ATR), scanning electron microscope–energy-dispersive X-ray (SEM-EDX) spectroscopy, thermal gravimetric analysis (TGA), tensile test, and density test. Successfully fabricated samples were tested before and after aging. The highest elongation at break of PVC flat sheet (244.96%) was obtained with the use of DOA plasticizer. The highest tensile strength was measured as 17.85 MPa for the sample containing 50 phr DOTP. Furthermore, no significant mass loss was observed up to 238 °C, while substantial decomposition occurred in the samples containing 50 phr DOTP, 50 phr DOA, and 50 phr TOTM between 238–338 °C, followed by gradual degradation at 483 °C and 683 °C. As a result, it has been determined that the use of DOA plasticizer in PVC compounds used in the cable industry is more effective than DOTP and TOTM plasticizers.

## 1. Introduction

Poly (vinyl chloride) (PVC) is one of the most important commercial thermoplastic polymers in the plastics industry [1,2] and is widely used in construction, packaging, automotive components [3,4], electrical cables, toys, pipes, and medical applications [5,6]. The most important reasons why PVC is preferred are its low production cost biocompatibility [7,8], optical transparency, inherent flexibility [9,10], long service life and compatibility with sterilization processes, and high chemical stability [11,12]. Compared to other commercial polymers [13,14], PVC compounds require a wide variety of additives during the extrusion process, including heat stabilizers, plasticizers, lubricants, fillers, processing aids, pigments, antioxidants, and UV stabilizers, collectively contributing to thermal stability, processability, and long-term performance [15,16]. One of the most important components in PVC compound production is plasticizers [17,18]. In recent years, a wide variety of synthetic and bio-based plasticizers are commercially available on the market [19,20]. Plasticized PVC is extensively utilized in the electrical industry owing to its low density, excellent dielectric properties, cost–performance ratio, high mechanical strength, and favorable rigidity, which collectively make it suitable for insulation and sheathing applications [21,22]. As the molecular weight of plasticizers increases [23], their persistence properties (volatility and extraction with various media) and ability to withstand degradation from ultraviolet (UV) light also increase [24,25]. Ma et al. [26] reported that nanoparticles such as functionalized ZnO nanoparticles could be well dispersed in PVC, and it significantly enhanced anti-UV capability. The preferred PVC composites have shown potential for environmentally sustainable applications in the UV protection field and low cost.

Addition of plasticizer enhances the flexibility and processability of PVC [27,28] by weakening intra- and intermolecular secondary bonding forces between polymer chains, thereby increasing free volume and reducing the glass transition temperature (Tg) of the material [29,30]. Dioctyl phthalate (DOP) esters are among the earliest developed and most widely employed plasticizers for PVC, providing effective plasticization through strong polymer–plasticizer interactions [31,32]. Due to increasing evidence and concerns regarding the potential health risks such as carcinogenicity and endocrine disruption effects of DOA, an increasing number of alternative non-phthalate plasticizers have been developed to replace traditional plasticizers for PVC compounds has been limited. Therefore, chemicals such as terephthalates, adipates and sebasates are used as plasticizers in PVC production. Jung et al. [33] reported that five selected alternative plasticizers of terephthalates, adipates, trimellites, citrates, and cyclohexane, which is dicarboxylic acids, bis(2-ethylhexyl) terephthalate (DEHTP), bis(2-ethylhexyl) adipate (DEHA), tris(2-ethylhexyl) benzene-1,2,4-tricarboxylate (TOTM), tributyl O-acetylcitrate (ATBC), and di(isononyl) cyclohexane-1,2-dicarboxylate (DINCH), respectively, have increasingly replaced conventional phthalates in many applications with potential endocrine health effects.

Dioctyl terephthalate (DOTP), which has a chemical structure like DOP, is widely used in the fabrication of PVC products due to its high compatibility with the PVC matrix, effective plasticization performance, low volatility, and wider acceptance compared to traditional phthalate plasticizers [34]. The effect of the plasticizer is controlled by several variables, such as the type of polymer, type of plasticizer, and thermal stability at processing temperature [35]. Altındag and Akdogan [36] investigated the migration of five non-phthalate plasticizers; DOTP, DOA, tributyl trimelliate (TBTM), TOTM, and dioctyl succinate with three levels (40, 60, and 80 phr) in PVC-based artificial leather. According to spectrophotometry results, DOTP has high compatibility with PVC because of having high interaction with PVC chains. Wang et al. [37] investigated thermal degradation kinetics of plasticized PVC with various plasticizers, including DOTP, DOP, acetytributyl citrate (ATBC), acetyl trioctyl citrate (ATOC), TOTM, and isosorbide ester (ID-37). Among these formulations, plasticized PVC with the addition of TOTM and plasticized PVC with the addition of DOTP exhibited onset degradation temperatures approximately 7 °C higher than those of the other plasticized PVC samples. In addition, plasticized PVC with addition of DOTP exhibited thermal stability that was superior to or comparable with that of plasticized PVC with addition of DOP. For example, di (2-ethylhexyl) adipate is a plasticizer frequently used in PVC formulations for cold-climate applications due to its low freezing point and excellent low-temperature flexibility [38]. DOA is well-known additive with chemical resistance and thermal resistance that does not decompose under heat for a long time. Guo-min et al. [39] reported that the rheological properties of DOTP plasticized plastisol were due to its good solubility properties. DOA has several disadvantages; it does not persist in the PVC matrix and has poor compatibility with PVC [40]. The polar group in the structure of the most common plasticizers are attracted to the polar C-Cl bond in PVC by dipole forces. This interaction weakens the C=O double bond in the plasticizers, and the stronger the attractive force between molecules, the weaker this bond becomes. This indicates plasticizer compatibility [41]. TOTM has many advantages such as low temperature, good chemical resistance and excellent low temperature performance for cable and high-temperature applications for most automotive applications [42]. However, it has a relatively high cost and limited availability. For these reasons, TOTM is combined with phthalates or polymeric plasticizers in some applications. Selecting low-cost plasticizers may result in poor economic outcomes, whereas considering the cost–performance balance is more appropriate for achieving cost efficiency [43]. Instead, the plasticizer dosage can be reduced by switching to a more effective plasticizer. There are many plasticizers in the literature, but the plasticizers used in PVC matrix in the cable industry are limited. The main reason for this situation is that plasticizers affect the cost [44]. Plasticizers play a crucial role in determining the mechanical properties of PVC while also significantly influencing the overall material cost [45].

DOTP, DOA, and TOTM have been investigated individually in the literature [18,29,45]. The aim of this study is to evaluate the efficiency of plasticization of these plasticizers as a function of their molecular structure, including branching, molecular weight, and end-group functionality, as well as their effects on mechanical performance and the retention of material properties during aging. The novelty of this study lies in the systematic comparison of these plasticizers, both individually and in binary combinations, with respect to their effects on the structural, morphological, thermal, and mechanical properties of PVC compounds for cable insulation applications before and after aging.

In this study, different plasticizers, namely DOTP, DOA, and TOTM, were incorporated into PVC compound formulations intended for cable insulation applications. The influence of plasticizer type on the structural, morphological, mechanical, and thermal properties of the prepared PVC flat-sheet samples was investigated in detail. Furthermore, the compatibility of the plasticizers with PVC was evaluated and compared using formulations containing each plasticizer individually as well as in binary combinations.

## 2. Materials and Methods

### 2.1. Materials

All chemicals were supplied by Plastifay Chemistry Inc. (Istanbul, Turkey) and were used as received without further purification. The physicochemical properties of the used plasticizers such as dioctyl adipate (DOA), tris(2-ethylhexyl) benzene-1,2,4-tricarboxylate (Plast TT), and dioctyl terephthalate (PLASTSOFT) are presented in Table 1. Poly (vinyl chloride) (PVC K-70), produced by suspension polymerization, was used as the polymer matrix. Calcite (CaCO_3_) with an average particle size of 2.00 µm and a density of 2.70 g/cm^3^ (ANDCARB CT-2X) was used as an inorganic filler. Stearic acid (C_18_H_36_O_2_, 18/65 grade), was used as a lubricant while a white powder calcium–zinc (Ca–Zn) stabilizer (ZER Chemical B222, Plastifay Chemistry Inc. (Istanbul, Turkey)) was used as a thermal stabilizer. Therefore, the K-value of the PVC, molecular weights and density of the plasticizers used in this study has been added according to the manufacturer’s technical data sheet (TDS).

### 2.2. Preparation of PVC Compounds

**Figure 1:**
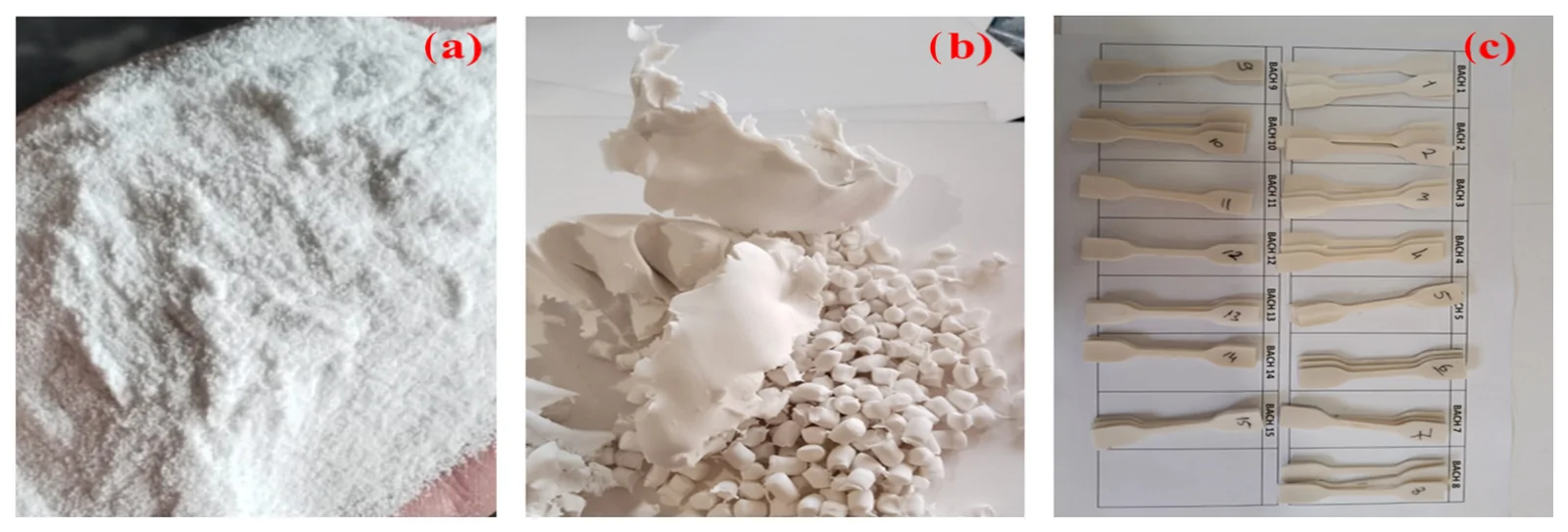
Process stages in sample preparation (**a**) mixture in powder form after the mixer; (**b**) PVC compound form after the extruder machine; (**c**) PVC flat sheet samples suitable for tensile testing.

DOTP, DOA, and TOTM additives were used as plasticizers while preparing the PVC compounds formulation for cable insulation applications. The formulation of PVC compounds is given in Table 2. PVC compounds were produced with optimum production parameters. The PVC formulations were firstly dry-mixed in a Dersan brand high-capacity mixer (Istanbul, Turkey; 500 kg capacity) at temperatures up to 100 °C (Figure 1a). After mixing in the mixer, the prepared PVC formulation mixture was kept in the cooling tank until it reached room temperature. After the cooling process, PVC compounds were prepared at 150 °C using a Takımsan brand granulation machine (Istanbul, Turkey) (Figure 1b). The PVC compounds produced are manufactured in flat sheet form and are used as insulation material in cable sheathing processes. Flat sheets were prepared from the formulated PVC compounds using a Takımsan brand mini extruder (Istanbul, Turkey) (Figure 1c). In this study, a planetary extruder with an L/D ratio of 28 was used. A temperature profile of 140–160 °C and an extruder screw speed of 21 rpm were used.

**Figure 2:**
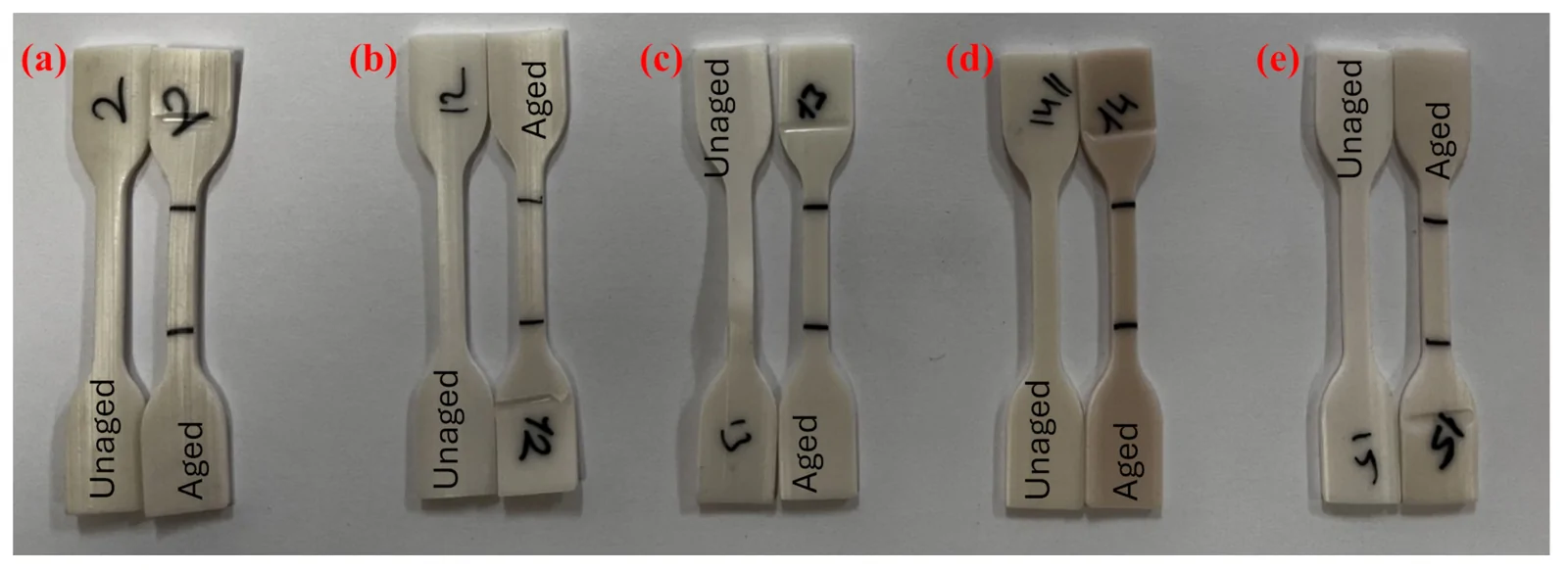
Comparison of color changes in the fabricated PVC flat sheet samples after the aging process: (**a**) Batch-2; (**b**) Batch-12; (**c**) Batch-13; (**d**) Batch-14; (**e**) Batch-15.

A thermal aging process was carried out to investigate the long-term effects of plasticizers on PVC for cable insulation applications. The prepared PVC flat sheet specimens for tensile test were thermally aged at 80 °C for 168 h in a BINDER M 115 model oven (Tuttlingen, Germany). The aging procedure was conducted in accordance with the EN 60811 standard [46]. During the thermal aging process, the PVC flat sheet undergoes degradation due to the rearrangement of its molecular chains induced by heat. The unaged and aged PVC flat sheet tensile test specimens are given in Figure 2.

### 2.3. Characterization

The effect of plasticizer on the structural properties of PVC flat sheet samples to be used in cable insulation production was examined by Fourier transform infrared-attenuated total reflectance (FTIR-ATR). A Perkin Elmer Spectrum-2 model FTIR-ATR device (Waltham, MA, USA) was used in FTIR analyses. As a result of the analysis, spectra were obtained in the wavelength range of 450–4000 cm^−1^.

The effect of plasticizer on the morphological properties of PVC flat sheet samples to be used in cable insulation production was examined by scanning electron microscope–energy-dispersive X-ray (SEM-EDX) spectroscopy analysis. Aged and unaged PVC flat sheet samples were examined by a JEOL 6610 model SEM device (Tokyo, Japan). During the SEM analysis, the fractured surface was examined after the tensile test. Before the SEM analysis, all PVC flat sheet samples were coated with gold.

**Figure 3:**
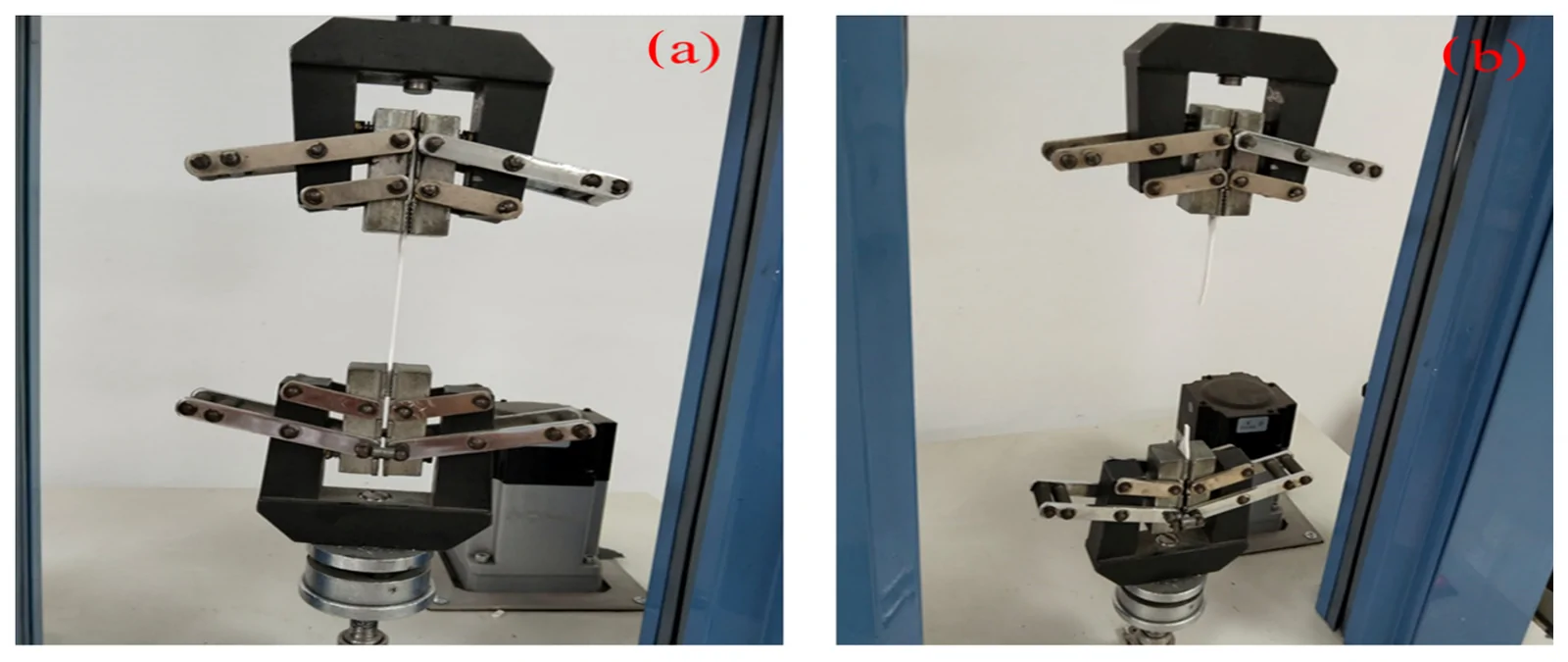
(**a**) Initial stage of tensile test of a PVC flat sheet sample; (**b**) a fractured PVC flat sheet sample after tensile testing.

The effect of plasticizer on the mechanical properties of aged and unaged PVC flat sheet samples to be used in PVC cable insulation production was examined by tensile test. The fabricated aged and unaged PVC flat sheets samples (Batch-2, Batch-12, Batch-13, Batch-14 and Batch-15) are given in Figure 2. The tensile test was performed in accordance with TS EN 60811 standard [46]. A Devotrans tensile test device (Istanbul, Turkey) was used during the tensile test as shown in Figure 3.

The effect of plasticizer on the thermal properties of PVC flat sheet samples to be used in PVC cable insulation production was examined by thermal gravimetric analysis (TGA). Thermal behaviors of PVC flat sheet samples were taken by an EXSTAR 6300 model SII NanoTechnology TGA device (Tokyo, Japan). TGA was carried out under nitrogen (N_2_) atmosphere from 25 to 1000 °C at a heating rate of 10 °C/min. As a result of the analysis, thermograms were obtained in the temperature range of 25 to 1000 °C.

Density test measurements of PVC flat sheet samples were performed by a Precisa xb 220a model analytical balance device (Precisa, Dietikon, Switzerland) according to ASTM D 792 standard [47].

## 3. Results

### 3.1. Results of FTIR Analysis

**Figure 4:**
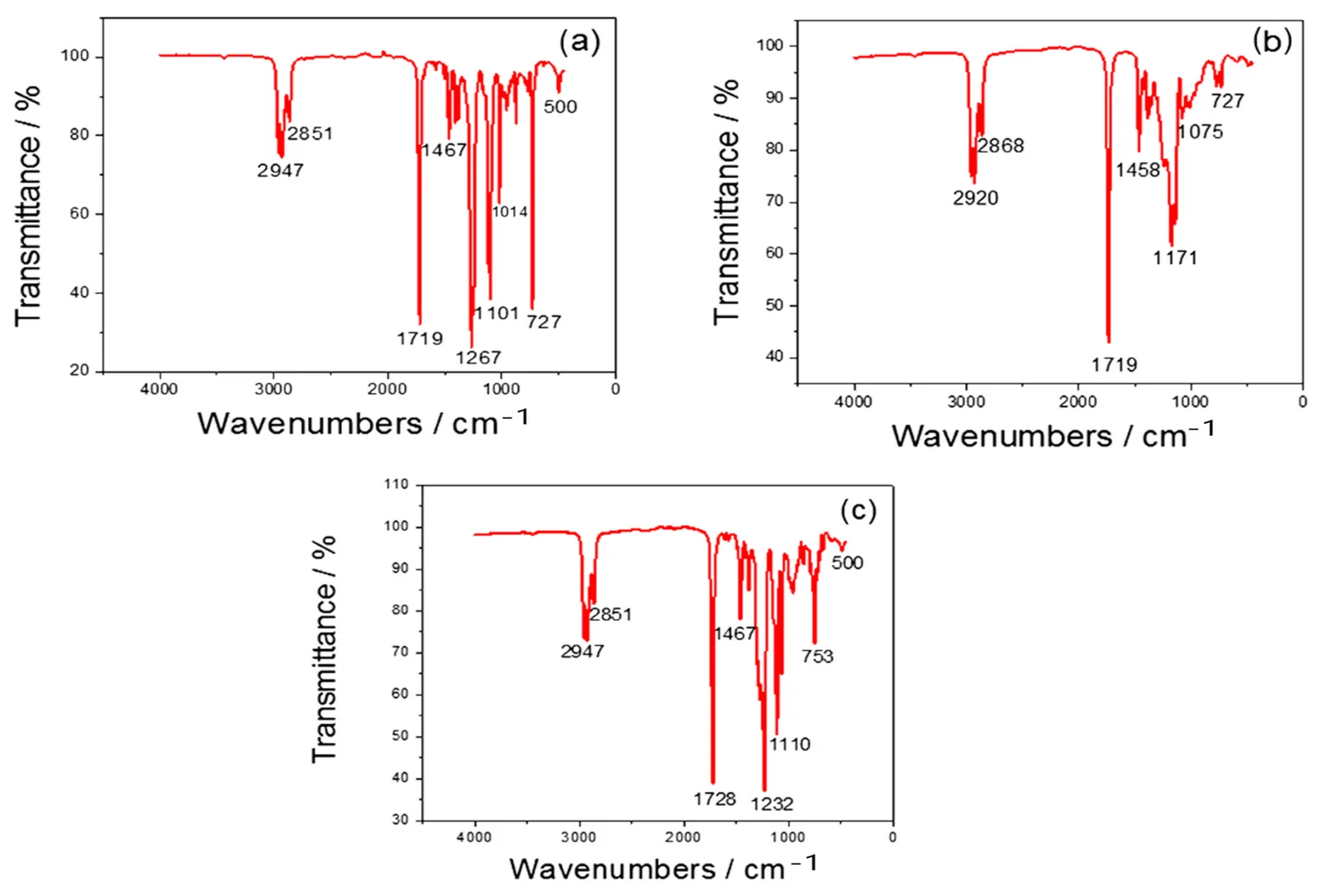
FTIR spectra of plasticizers of (**a**) DOTP; (**b**) DOA; (**c**) TOTM.

FTIR spectra of PVC samples plasticized with DOTP (C_24_H_38_O_4_) (Figure 4a), DOA (C_24_H_38_O_4_) (Figure 4b), and TOTM (C_33_H_54_O_6_) (Figure 4c) were obtained to better understand the interactions between different plasticizer types and PVC chains. The characteristic peaks of the PVC flat sheet are observed in the 600–700 cm^−1^ region, which corresponds to the fingerprint region of the spectrum. The band at 1710–1735 cm^−1^ belongs to the C=O stretching vibrations in the carboxyl group overlapping the ester group. FTIR spectra of PVC have been discussed in different studies in the literature [48]. As shown in Figure 4, all types of adipate-based plasticizers contain carbonyl functional groups. The trimellitate-based plasticizer TOTM exhibits two types of C=O groups: one corresponding to free carbonyl groups and the other associated with dipole–dipole interactions.

**Figure 5:**
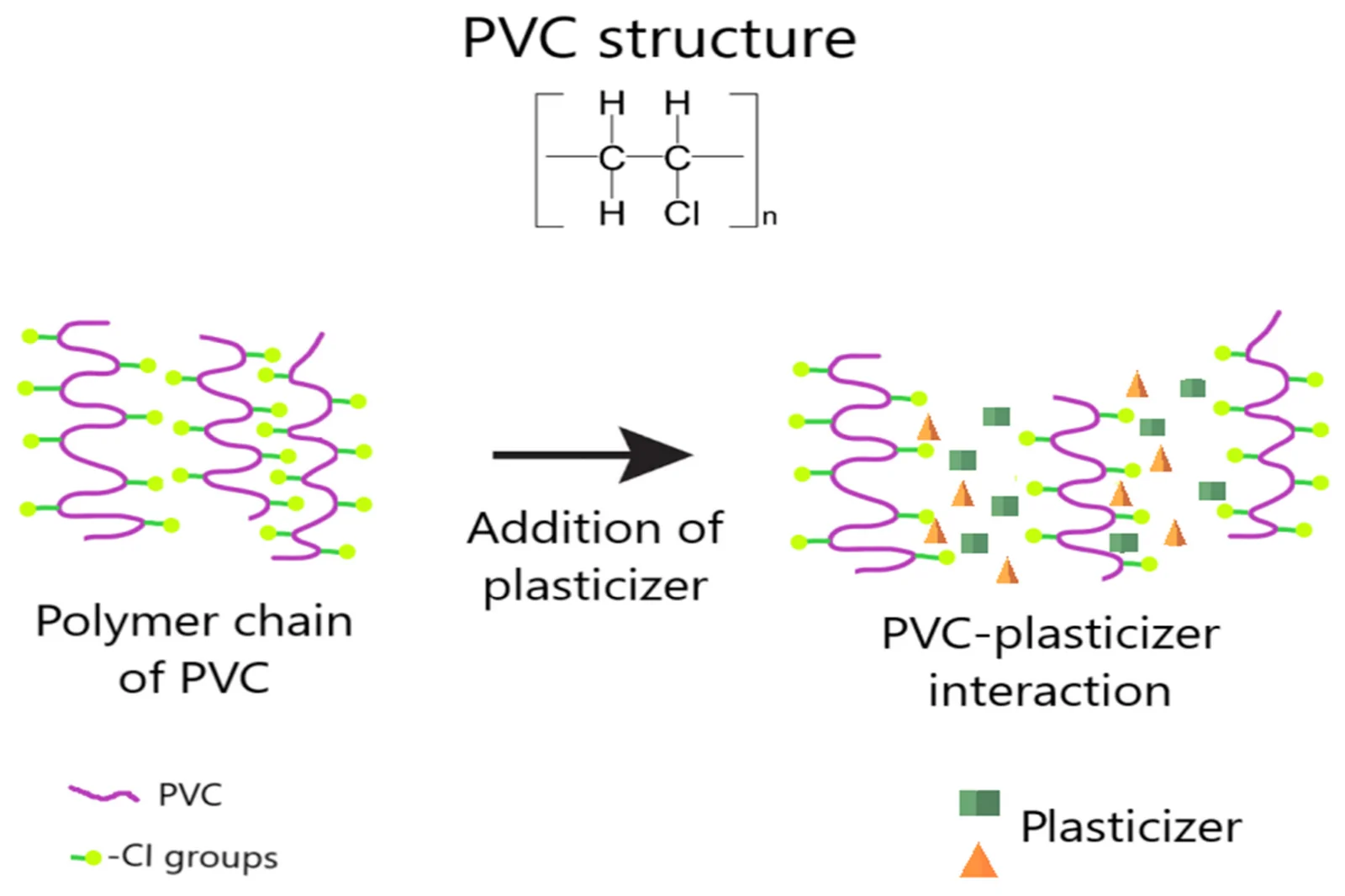
Schematic illustration of the interactions between PVC chains and different plasticizers.

PVC flat sheet sample containing DOTP at 1719 cm^−1^, PVC flat sheet sample containing DOA at 1754 cm^−1^ and PVC flat sheet sample TOTM at 1728 cm^−1^ indicates the carbonyl group (C=O) ester plasticizer in the granules. It is also attributed to C=O stretching and bending vibrations in the aromatic ring between 1450–1500 cm^−1^ whereas those around 700–750 cm^−1^ correspond to characteristic C–Cl vibrations and aromatic ring modes. FTIR spectra of all plasticizers (DOTP, DOA, and TOTM) show the same trend in PVC flat sheets. DOA generally has higher peak lengths than the phthalate group. DOTP almost does not show peak change with heat treatment [48]. DOA exhibited a sharper and more intense carbonyl band, indicating higher molecular mobility and plasticization efficiency. Differences in the position and intensity of the carbonyl bands indicate that the molecular structure of the plasticizer influences its interaction with PVC and its plasticization behavior. The interactions between PVC chains and plasticizers are schematically illustrated in Figure 5.

### 3.2. Results of Density Test

Plasticizers with higher molecular weights and aromatic structures generally exhibit higher densities leading to an increase in PVC compound density. The density values of the PVC flat-sheet samples were found to be consistent with the molecular weights of the used plasticizers which are DOA, DOTP, and TOTM. Among the samples (Batch-2, Batch-11, Batch-12, Batch-13, Batch-14, and Batch-15), the PVC flat sheet containing 25 phr DOTP and 25 phr DOA plasticizers exhibited the highest distribution and density (1.444 g/cm^3^) for Batch-14. Conversely, Batch-11 exhibited the lowest density (1.418 g/cm^3^) with 50 phr DOA. At an equivalent plasticizer content (50 phr), the densities of Batch-2 (DOTP), Batch-11 (DOA), and Batch-12 (TOTM) were 1.441, 1.418, and 1.443 g/cm^3^, respectively. The density values were measured as 1.441, 1.418, 1.443, 1.430, and 1.425 g/cm^3^ for Batch-2, Batch-11, Batch-12, Batch-13, and Batch-15, respectively. The plasticizer including the molecular weights of plasticizers is given in Table 1. The PVC flat sheet sample containing DOTP has a molecular weight of 390 g/mol and its density is obtained as 1.441 g/cm^3^. In the DOA component PVC sample, the lowest molecular weight was 370 g/mol and the lowest density (1.418 g/cm^3^). The molecular weight of plasticizers significantly influences the physical and mechanical properties of PVC compounds. Therefore, the observed density variations can be attributed to differences in the molecular structure, molecular weight, and compatibility of the plasticizers with PVC.

### 3.3. Results of SEM Analysis

**Figure 6:**
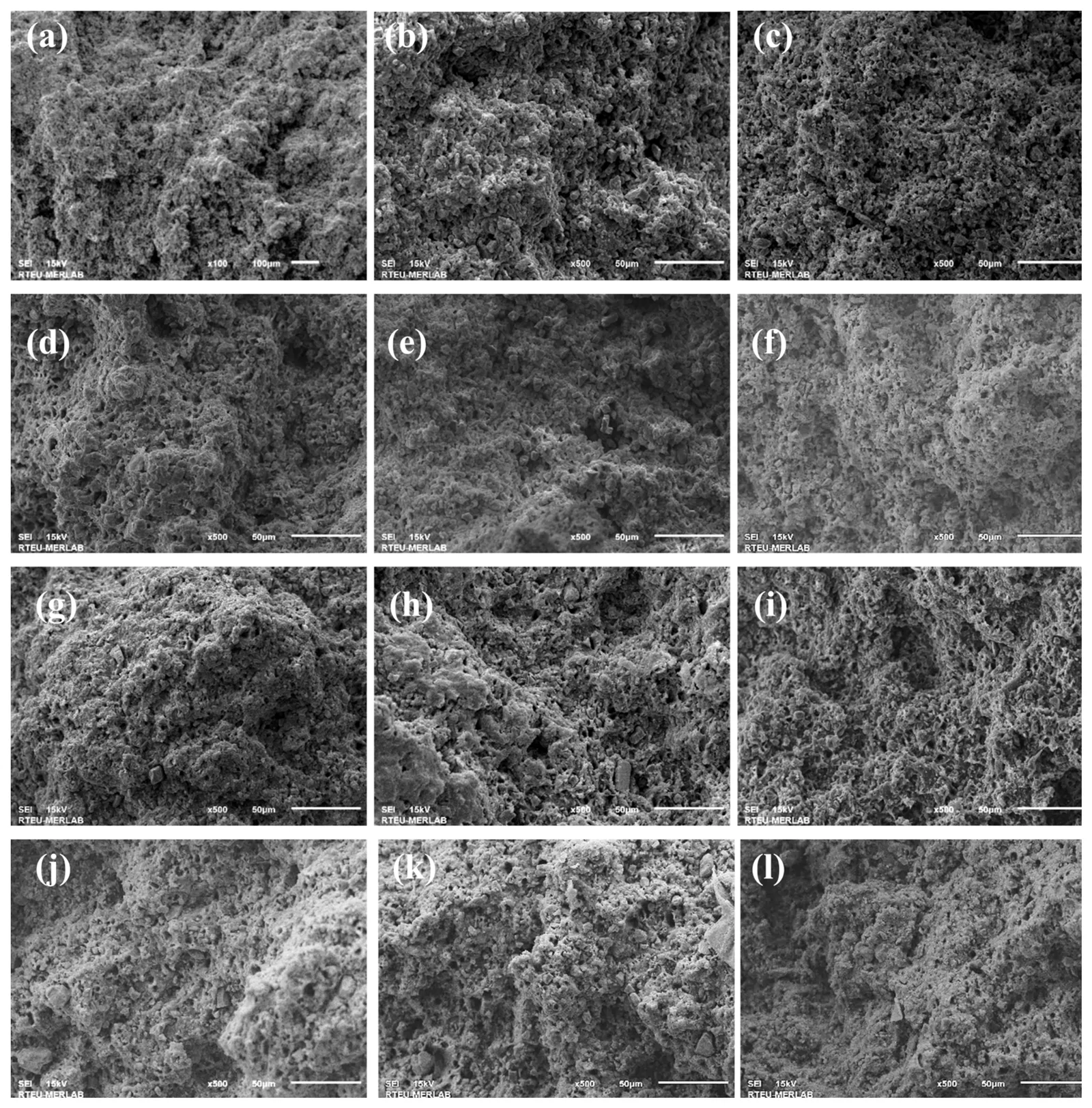
SEM micrographs of PVC flat sheet samples of (**a**) unaged Batch-2; (**b**) unaged Batch-11; (**c**) unaged Batch-12; (**d**) aged Batch-2; (**e**) aged Batch-11; (**f**) aged Batch-12; (**g**) unaged Batch-13; (**h**) unaged Batch-14; (**i**) unaged Batch-15; (**j**) aged Batch-13; (**k**) aged Batch-14; (**l**) aged Batch-15.

The effects of different types of plasticizers on morphology, SEM micrographs comparison of fractured surfaces of after aged and unaged PVC flat sheet samples after tensile tests are given in Figure 6. Due to the difference in chemical structure, there was a difference in compatibility between plasticizers and PVC chains, resulting in different microstructures in SEM micrographs. Different amounts of pores occurred in PVC flat sheet samples depending on the types of plasticizers. The efficiency of plasticizers might contribute to the distribution of molecules into PVC chains when an external force is applied to the PVC flat sheet samples (Figure 6) [49].

The formation of pores causes the PVC flat sheet samples to be susceptible to damage due to a load. Additionally, when aged and unaged samples are compared, the SEM micrographs are different with each other. Pore formation, segmental separation, and irregular surfaces were observed in PVC flat sheet samples after aging. The aging process resulted in the dehydrochlorination of the C-Cl bond in the structure of PVC and the formation of conjugated double bonds and polyene sequences [50]. These changes lead to undesirable color changes (Figure 2) and deterioration of the surface and mechanical properties of the PVC flat sheet samples. Aged PVC flat sheet samples have different micro-pores depending on the types of plasticizers. So, the overall structure of aged PVC flat sheet samples is more stable and durable compared to unaged PVC flat sheet samples. Moreover, in unaged PVC flat sheet samples, surface integrity is high, the morphology is homogeneous. However, porosity is low. This is because pore formation without aging increases the mobility of PVC chains. Mohammed et al. [51] reported that plasticizers are incorporated into composite materials to improve the flexibility of starch-based bio-composites and to reduce pore formation within the composite structure.

### 3.4. Results of Tensile Test

Tensile tests were performed on PVC flat sheet samples before and after the aging process. The effects of different plasticizer types on the mechanical properties were characterized by comparing the tensile strength and elongation at break values of aged and unaged PVC flat sheet samples. Plasticizers play an important role in enhancing both the tensile strength and elongation at break of PVC flat sheet samples for cable insulation. In this study, tensile test results of PVC flat sheet samples are given in Table 3. The highest elongation at break in the presence of plasticizers in PVC was obtained as 244.96% for DOA, 217.67% for DOTP, and 207.14% for TOTM.

The effect of plasticizer types on the tensile strength of unaged and aged PVC flat sheet samples is given in Table 3. The highest tensile strength was obtained as 16.97 MPa (Batch-12, 50 phr TOTM). However, the lowest tensile strength was indicated as 12.28 MPa for DOA containing PVC flat sheet samples. This can be explained by the compatibility of the chemical structure of plasticizers with PVC chains. Due to the absence of aromatic rings of DOA, its synergistic plasticizing effect is weak compared to DOTP and TOTM. This is mainly because the presence of ester groups, alkane chains, and aromatic groups in DOTP and TOTM structures has a positive effect on weakining the interactions between PVC chains and plasticized PVC [37,52].

In addition, when elongation and flexibility are considered, Batch-11, corresponding to the PVC compound plasticized with DOA, exhibited the highest elongation at break, with a value of 244.96%. Plasticizers modify the elongation behavior of PVC flat sheet samples by weakening the intermolecular attractions between polymer chains. In the presence of plasticizers, less energy is required for the movement of PVC chains. Among the prepared PVC compounds, those containing TOTM showed the lowest elongation at break. The addition of DOA as a plasticizer significantly increased the flexibility of the PVC chains, resulting in higher elongation at break values for the PVC flat sheet samples. The size and molecular structure of the plasticizer influence its ability to move freely within the PVC matrix. The linear molecular structure of the DOA plasticizer does not hinder the movement of PVC chains during stretching, thereby providing greater flexibility.

The chemical structure of DOA is characterized by long, linear hydrocarbon chains, whereas PVC chains and the DOTP and TOTM plasticizers contain bulkier functional groups, such as aromatic rings. A higher plasticization efficiency in PVC flat sheet samples generally results in increased elongation at break and reduced tensile stress. Furthermore, the lower resistance to PVC chain mobility during tensile deformation leads to a decrease in the tensile strength of the flat sheet samples. Experimental results showed that the tensile strength of the PVC flat sheet samples decreased after the aging process (Table 3), suggesting that thermal exposure-initiated degradation of the PVC chemical structure. A similar reduction in elongation at break was also observed following aging. Overall, the SEM micrographs are consistent with the mechanical test results.

**Figure 7:**
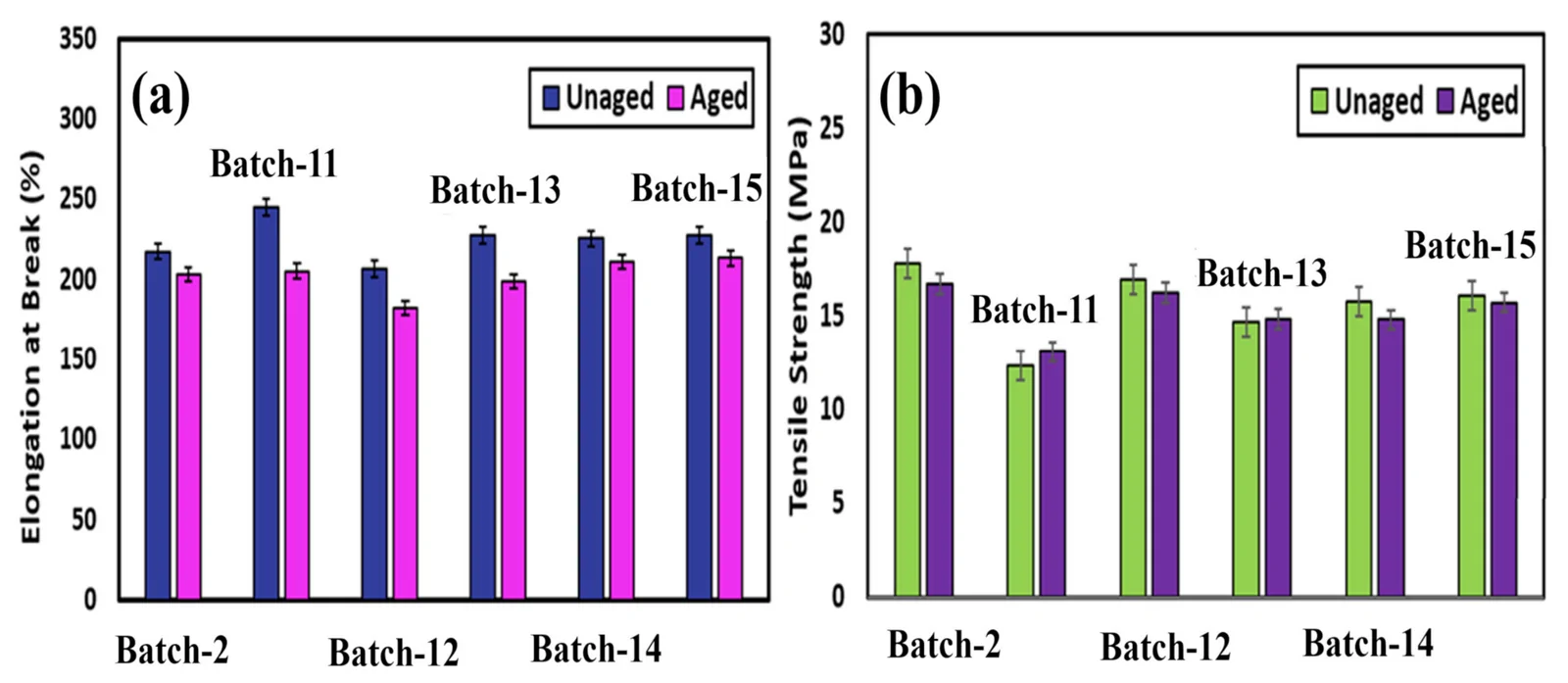
(**a**) Elongation at break of Batch-2, Batch-11, Batch-12, Batch-13, Batch-14, and Batch-15 of aged and unaged PVC flat sheet samples; (**b**) tensile strength of Batch-2, Batch-11, Batch-12, Batch-13, Batch-14, and Batch-15 of aged and unaged PVC flat sheet samples.

**Figure 8:**
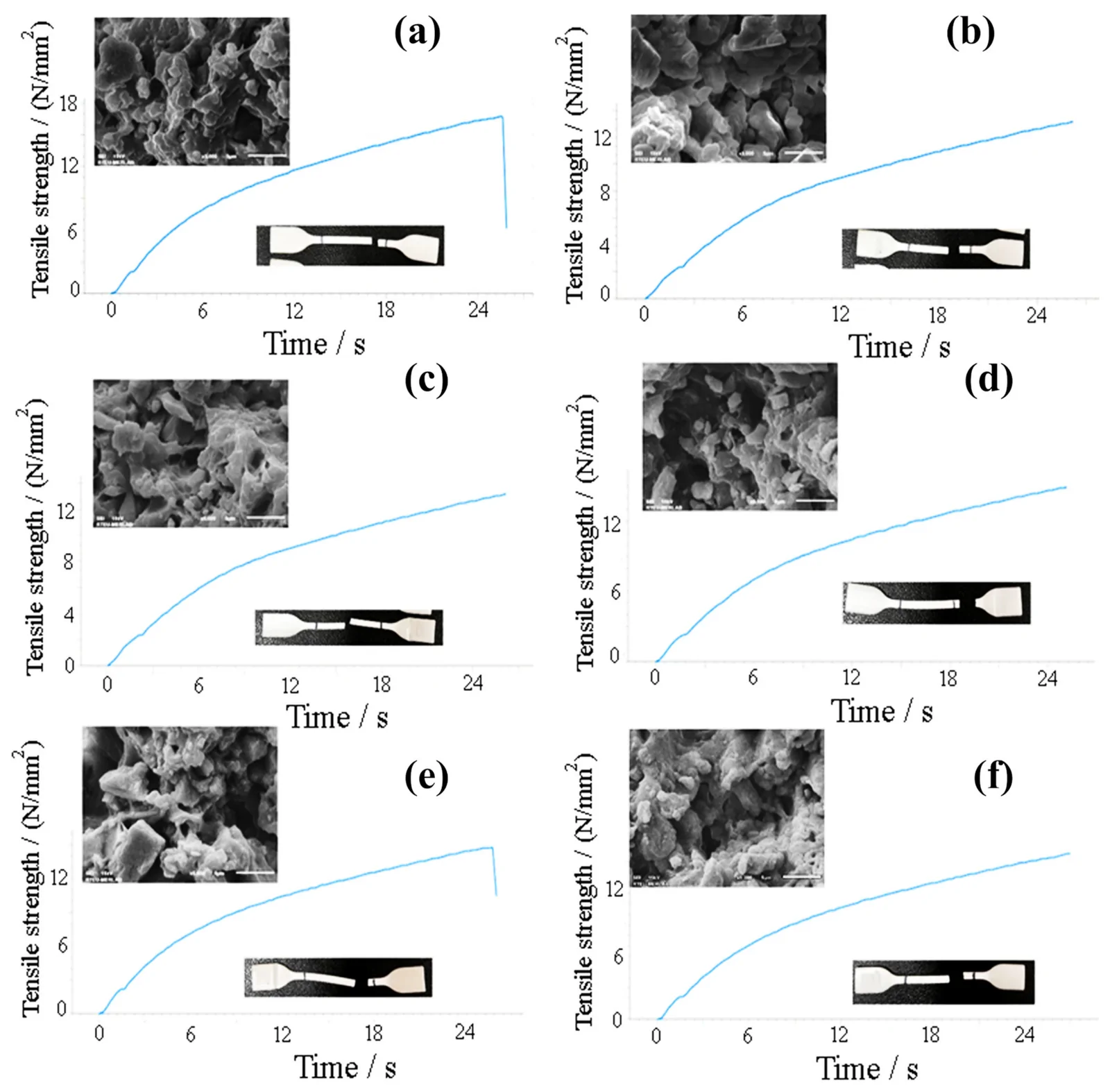
Comparison of SEM micrographs and tensile strength–time curves of aged PVC flat sheet samples of (**a**) Batch-2; (**b**) Batch-11; (**c**) Batch-12; (**d**) Batch-13; (**e**) Batch-14; (**f**) Batch-15.

Elongation at break and tensile strength (Batch-2, Batch-11, Batch-12, Batch-13, Batch-14, and Batch-15) of aged and unaged PVC flat sheet samples are given in Figure 7. The elongation at break decreased in Batch-2, Batch-11, Batch-12, Batch-13, Batch-14, and Batch-15 after the aging process compared to the unaged process (Figure 7a). However, the tensile strength increased slightly in Batch-11 and Batch-13 compared to the aging process. Thus, the tensile strength decreased in Batch-2, Batch-12, Batch-14, and Batch-15 (Figure 7b). We obtained similar results in comparison with the literature. Yang et al. [53] synthesized four isosorbide diesters: isosorbide dibutyrate (SDB), isosorbide didecanoate (SDD), isosorbide dihexanoate (SDH), and isosorbide dioctanoate (SDO), as biobased plasticizers and DOTP to comparison were blended with PVC which is 10 g of PVC, 4 g of plasticizer and 0.4 g of calcium zinc heat stabilizer. Elongation at break were evaluated as 238.3 ± 2.2%. The effects of different types of plasticizers and their combinations on morphology are shown in Figure 8. There are undesirable color changes and quantities of the PVC flat sheet samples due to the aging process. Thus, there is a decrease in the mechanical performance of the PVC flat sheet samples from SEM micrographs due to aging effects and deformation behavior. Comparisons of fracture surface SEM micrographs of aged and unaged PVC flat sheet samples after the tensile tests are given in Figure 8. The influence of plasticizer molecular weight was verified through changes observed in mechanical properties. Low-molecular-weight plasticizers diffuse more readily between polymer chains, resulting in pronounced changes in polymer properties. A homogeneous and smooth surface morphology indicates good plasticizer dispersion and enhanced plasticity, whereas the presence of pores, voids, or phase-separated regions suggests reduced compatibility between the plasticizer and PVC leading to a deterioration in mechanical properties such as tensile strength and elongation at break. Conversely, uniform microstructure generally shows improved mechanical performance due to more effective plasticization and stronger PVC chain–plasticizer interactions. TOTM’s ester structure enhances PVC interactions, reducing volatility and migration while improving long-term mechanical stability [10].

### 3.5. Results of TGA Analysis

**Figure 9:**
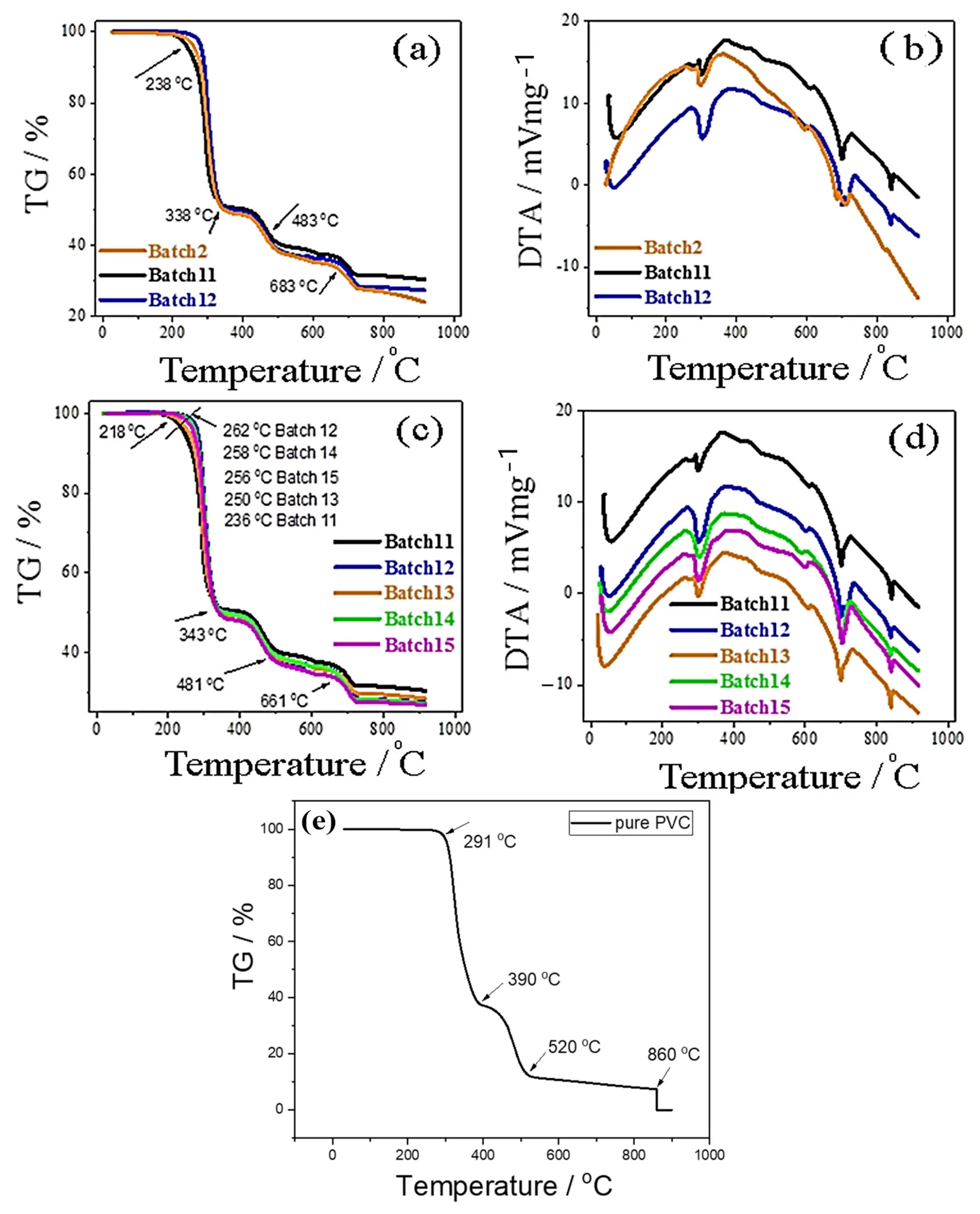
(**a**) TG-Temperature plot for Batch-2, Batch-11, and Batch-12; (**b**) DTA-Temperature plot for Batch-2, Batch-11, and Batch-12; (**c**) TG- Temperature plot for Batch-11, Batch-12, Batch-13, Batch-14, and Batch-15; (**d**) DTA-Temperature plot for Batch-11, Batch-12, Batch-13, Batch-14, and Batch-15; (**e**) TG-Temperature plot for pure PVC.

The TGA thermograms indicate that the thermal degradation behavior of pure or non-plasticized and plasticized PVC flat sheet samples is significantly influenced by the type of plasticizers. The TG thermograms (thermogravimetric temperature plots) of the PVC flat sheet samples are given in Figure 9. TGA thermogram of pure PVC flat sheet sample was exhibited a two-step degradation process (Figure 9e). The onset degradation temperature of pure PVC was higher than that of plasticized PVC. Pure PVC consists of dehydrochlorination between 291 and 390 °C, followed by the degradation of carbonaceous residue and polyene structures between 390 and 520 °C. During the first stage of PVC thermal degradation, molecules attached to the polymer backbone are removed from the polymer chain, resulting in the formation of an unsaturated chain structure that subsequently undergoes further degradation [54,55].

Plasticized PVC flat sheet samples exhibited an additional initial weight-loss stage associated with the evaporation or decomposition of the plasticizer before the main PVC degradation occurs. The type of plasticizer significantly influences the thermal degradation behavior. It consists of 50 phr DOTP for Batch-2, 50 phr DOA for Batch-11, and 50 phr TOTM for Batch-12. The first sharp weight loss observed in the TGA curve corresponds to the elimination of HCl corresponds to the dehydrochlorination of PVC chains, whereas the subsequent weight losses are attributed to carbonaceous degradation. There is no significant mass loss up to 238 °C. Although it is very small value, the mass loss is caused by moisture and the ions of other foreign groups. After 238 °C, a significant mass loss occurred in Batch-2, Batch-11, and Batch-12 up to 338 °C (Figure 9a). Afterwards, the degradation of materials continued gradually by thermal degradation/pyrolysis with cyclization occurs at 483 and 683 °C [56].

In the differential thermal analysis (DTA) thermograms, endothermic behavior was observed up to 230 °C, followed by exothermic behavior at higher temperatures (Figure 9b). TG-temperature plots of Batch-11, Batch-12, Batch-13, Batch-14, and Batch-15 are given in Figure 9c. Batch-11, Batch-12, Batch-13, Batch-14, and Batch-15 samples contain PVC compound with 50 phr DOA, 50 phr TOTM, 25 phr DOTP and 25 phr TOTM, 25 phr DOTP and 25 phr DOA, 25 phr DOA and 25 phr TOTM, respectively. In the DTA thermograms, long and sharp peaks indicate rapid decomposition of the structure, whereas short peaks represent slower decomposition. Thermal decomposition of the above-mentioned plasticizer doping rates starts from 218 °C. Different decomposition temperatures at 262 °C for Batch-12, at 258 °C for Batch-14, at 256 °C for Batch-15, at 250 °C for Batch-13, and at 236 °C for Batch-11 were given in Figure 9c. Batch-12 (PVC with 50 phr TOTM component) sample has the highest thermal resistance and started to decompose at 262 °C. However, the Batch-11 (PVC with 50 phr DOA component) sample has the lowest thermal resistance and starts to decompose at 236 °C (Figure 9c). A similar DTA-temperature plot was given on Figure 9d. PVC flat sheet samples plasticized with DOA exhibit an earlier onset of thermal degradation compared to those containing DOTP and TOTM. This behavior can be attributed to the lower molecular weight and higher chain mobility of DOA. While the linear molecular structure of DOA enhances the mobility of PVC chains, the larger molecular structures and aromatic functional groups of DOTP and TOTM restrict PVC chain mobility. The TGA–DTA thermograms indicate that plasticizers with higher molecular weight and more complex molecular structures improve the thermal stability of PVC flat sheet samples. Liu et al. reported that [38] the initial degradation temperature of DOTP was higher than that of DOA, at about 20 °C higher than DOTP for PVC samples. Yang et al. [53] reported that DOTP exhibited better stability than bio-based plasticizer for PVC samples because of providing excellent thermal stability of the benzene ring.

## 4. Conclusions

In this study, PVC compounds for applications of cable insulation were developed using carbonyl-containing plasticizers with different molecular weights. In the mechanical tests, the highest tensile strength was measured as 17.85 MPa with the use of 50 phr DOTP in Batch-2. However, the tensile strengths decreased to 12.38, 16.97, 14.71, 15.77, and 16.09 MPa for Batch-11, Batch-12, Batch-13, Batch-14, and Batch-15, respectively. The highest reduction in elongation at break was obtained 182.56% from the adipate-based DOA plasticizer. This decrease in elongation at break was observed from 244.96% to 205.37% in DOA after aging test at 80 °C and 168 h. However, the other reduction in elongation at break were measured as 205.37% for DOTP and 203.59% for TOTM plasticizers. Plasticizers change the elongation behavior of PVC flat sheet samples by weakening the attraction between polymer chains. In the FTIR analysis, C=O stretching vibration peaks in the carboxyl group overlapping with the ester group were clearly present in all samples. The peaks are sharp and distinct because they are formed because of the esterification of plasticizers with phthalates and alcohols. There is no significant mass loss up to 238 °C due to the volatility of moisture and other foreign substances from TGA-DTA analysis. After 238 °C, a serious mass loss occurred in Batch-2, Batch-11, and Batch-12 up to 338 °C. Afterwards, the decomposition continued gradually by alcoholism at 483 and 683 °C. The lowest molecular weight and density were obtained as 370 g/mol and 1.418 g/cm^3^ for DOA plasticizer in the PVC flat sheet sample. In addition, in SEM micrographs, pore formation, segmental separation, and irregular surfaces were observed in PVC flat sheet samples after the aging process. As a result, DOA plasticizer is a more effective additive than TOTM and DOTP in PVC flat sheets to supply a synergistic effect for PVC cable insulation applications.

## Figures and Tables

**Table 1:** Properties of the plasticizers (DOA, TOTM, and DOTP) used in this study.

Properties of Plasticizer	Units	DOA	TOTM	DOTP
Molecular weight	g/mol	370	545	391
Density	g/cm^3^	0.927	0.984	0.980
Refractive index (at 20 °C)	-	1.446–1.448	1.483–1.487	1.490

**Table 2:** Chemical composition of PVC compounds.

Samples	PVC (phr)	Calcite (phr)	Stabilizer (phr)	Stearic Acid (phr)	Plasticizers (phr)		
					DOTP	TOTM	DOA
Batch-2	100	50	3	0.3	50	0	0
Batch-11	100	50	3	0.3	0	0	50
Batch-12	100	50	3	0.3	0	50	0
Batch-13	100	50	3	0.3	25	25	0
Batch-14	100	50	3	0.3	25	0	25
Batch-15	100	50	3	0.3	0	25	25

**Table 3:** Tensile test results of PVC flat sheet samples of aged and unaged samples.

	Aged		Unaged	
Samples	Tensile Strength (MPa)	Elongation at Break (%)	Tensile Strength (MPa)	Elongation at Break (%)
Batch-2	17.85	217.67	16.73	203.59
Batch-11	12.38	244.96	13.1	205.37
Batch-12	16.97	207.14	16.28	182.56
Batch-13	14.71	227.55	14.83	199.08
Batch-14	15.77	225.67	14.82	211.33
Batch-15	16.09	227.73	15.75	213.46

## Data Availability

The original contributions presented in this study are included in the article. Further inquiries can be directed to the corresponding author.
